# Supplementary material for: Efficacy of the Adjunct Use of Povidone-Iodine or Sodium Hypochlorite with Non-Surgical Management of Periodontitis: A Systematic Review and Meta-Analysis
Source: J Clin Med. 2022 Nov 7;11(21):6593. doi: 10.3390/jcm11216593 (PMC9658783; doi:10.3390/jcm11216593)
Supplement: Supplementary file 1 [file jcm-11-06593-s001.zip › Supplementary Table S1 .pdf]

Database Search

PubMed [Date of search: Sept 10, 2022]:

No	Search Query	Results
#1	Periodontitis OR peri-odontitis OR "Periodontitis"[Mesh]	121,354
#2	“sodium hypochlorite” OR NaOCl OR "Sodium Hypochlorite"[Mesh]	10,213
#3	Betadine OR “povidone iodine” OR povidone-iodine OR providine OR disadine OR isodine OR "Povidone-Iodine"[Mesh]	5,222
#4	#2 OR #3	15,266
#5	“nonsurgical periodontal treatment” OR “non-surgical periodontal treatment” OR “nonsurgical periodontal therapy” OR “non-surgical periodontal therapy” OR “nonsurgical treatment” OR “non-surgical treatment” OR “conventional treatment” OR “mechanical debridement” OR “subgingival irrigation” OR “scaling and root planing” OR “scaling root planing” OR "Root Planing"[Mesh]	25,045
#6	#1 AND #4 AND #5	73

Scopus [Date of search: Sept 9, 2022]:

No	Search Query	Results
#1	ALL (Periodontitis) OR ALL (peri-odontitis)	112686
#2	ALL (“sodium hypochlorite”) OR ALL (NaOCl)	39500
#3	ALL (Betadine) OR ALL (“povidone iodine”) OR ALL (povidone-iodine) OR ALL (providine) OR ALL (disadine) OR ALL (isodine)	25456
#4	#2 OR #3	64130
#5	ALL (“nonsurgical periodontal treatment”) OR ALL (“non-surgical periodontal treatment”) OR ALL (“nonsurgical periodontal therapy”) OR ALL (“non-surgical periodontal therapy”) OR ALL (“nonsurgical treatment”) OR ALL (“non-surgical treatment”) OR ALL (“conventional treatment”) OR ALL (“mechanical debridement”) OR ALL (“subgingival irrigation”) OR ALL (“scaling and root planing”) OR ALL (“scaling root planing”)	118494
#6	#1 AND #4 AND #5	650

Web of Science [Date of search: Sept 9, 2022]:

No	Search Query	Results
#1	ALL=Periodontitis OR ALL=peri-odontitis	35638
#2	ALL="sodium hypochlorite" OR ALL=NaOCl	12217
#3	ALL=Betadine OR ALL="povidone iodine" OR ALL=povidone-iodine OR ALL=providine OR ALL=disadine OR ALL=isodine	5277
#4	#2 OR #3	17362
#5	ALL="nonsurgical periodontal treatment" OR ALL="non-surgical periodontal treatment" OR ALL="nonsurgical periodontal therapy" OR ALL="non-surgical periodontal therapy" OR ALL="nonsurgical treatment" OR ALL="non-surgical treatment" OR ALL="conventional treatment" OR ALL="mechanical debridement" OR ALL="subgingival irrigation" OR ALL="scaling and root planing" OR ALL="scaling root planing"	24254
#6	#1 AND #4 AND #5	64

CENTRAL [Date of search: Sept 9, 2022]:

No	Search Query	Results
#1	Periodontitis OR peri-odontitis	6102
#2	"sodium hypochlorite" OR NaOCl	1087
#3	Betadine OR "povidone iodine" OR povidone-iodine OR providine OR disadine OR isodine	1984
#4	#2 OR #3	3040
#5	"nonsurgical periodontal treatment" OR "non-surgical periodontal treatment" OR "nonsurgical periodontal therapy" OR "non-surgical periodontal therapy" OR "nonsurgical treatment" OR "non-surgical treatment" OR "conventional treatment" OR "mechanical debridement" OR "subgingival irrigation" OR "scaling and root planing"	1683
#6	#1 AND #4 AND #5	16

Clinicaltrials.gov [Date of search: Sept 9, 2022]:

No	Search Query	Results
#1		
#2		
#3		
#4		
#5		
#6		

Google Scholar [Date of search: Sept 9, 2022]:

No	Search Query	Results
With all of the words	Periodontitis non surgical	
With the exact phrase		
With at least one of the words	“sodium chlorite” “povidone iodine” povidone-iodine NaOCl	
Total	As per the guidelines, only the 1st 100-200 records will be retrieved and screened	
