# Supplementary figures and images for: Efficacy of the Adjunct Use of Povidone-Iodine or Sodium Hypochlorite with Non-Surgical Management of Periodontitis: A Systematic Review and Meta-Analysis
Source: J Clin Med. 2022 Nov 7;11(21):6593. doi: 10.3390/jcm11216593 (PMC9658783; doi:10.3390/jcm11216593)

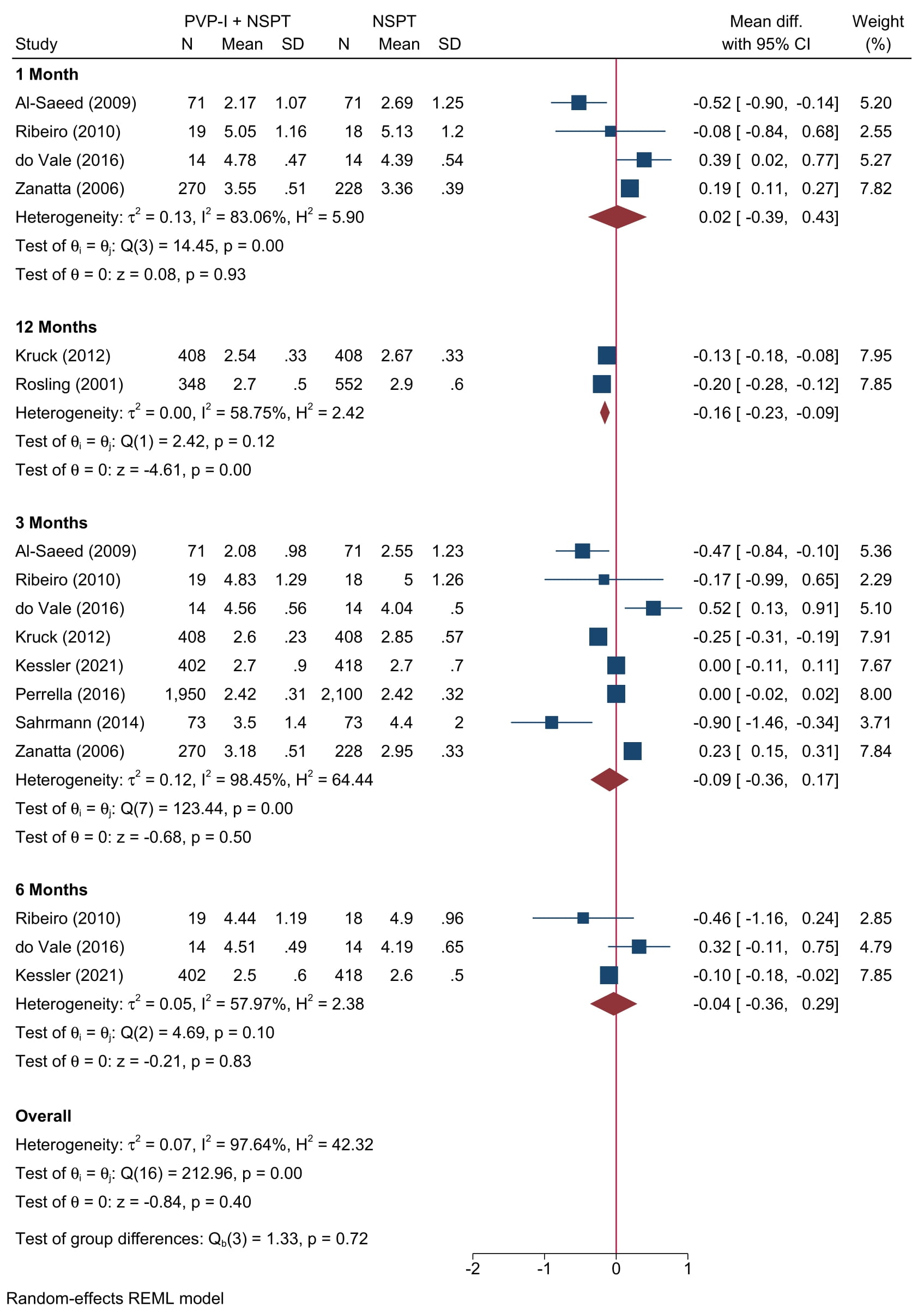

Supplement: Supplementary file 1 [file jcm-11-06593-s001.zip › Supp Figure S1.jpg]

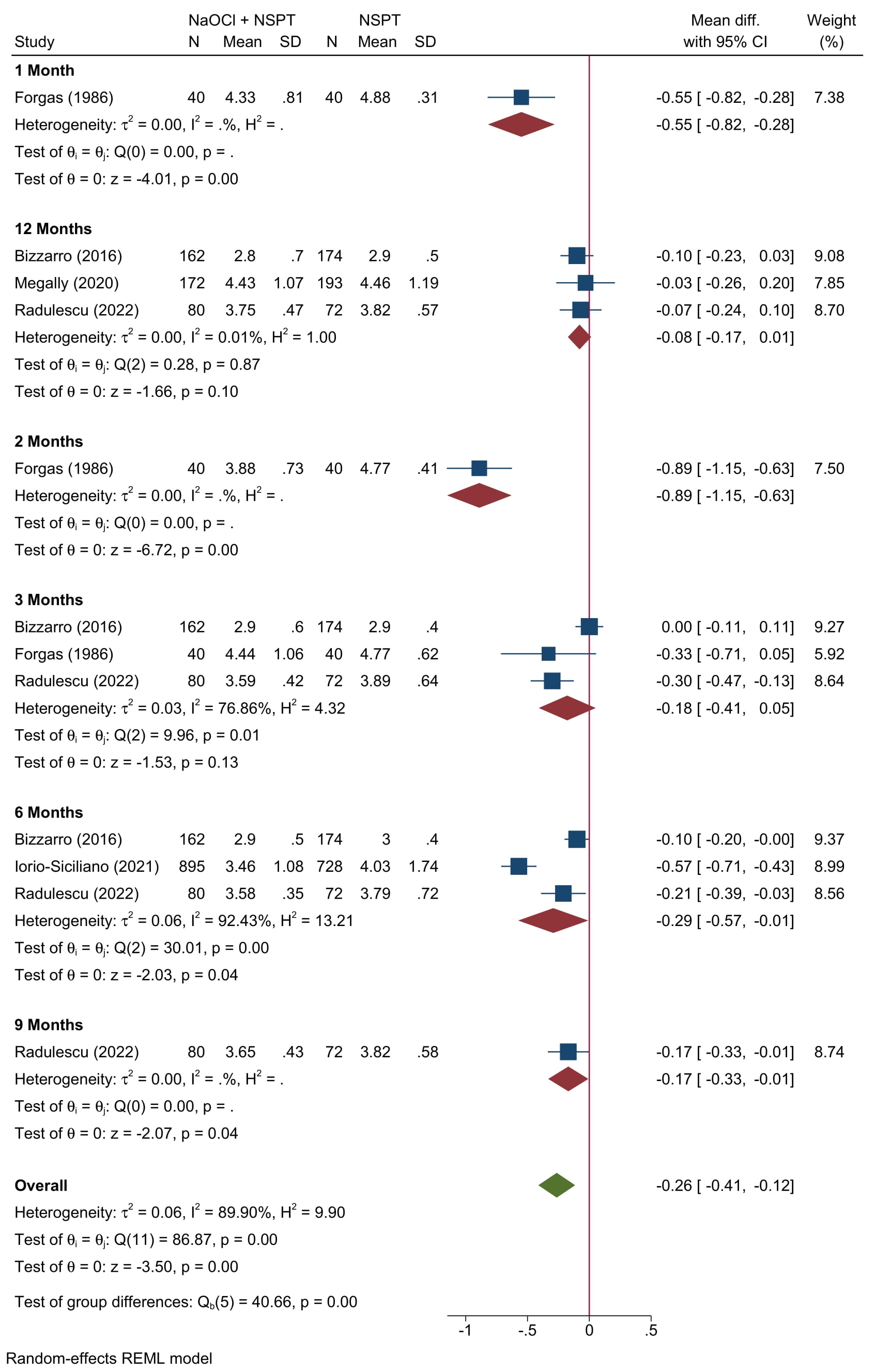

Supplement: Supplementary file 1 [file jcm-11-06593-s001.zip › Supp Figure S10.jpg]

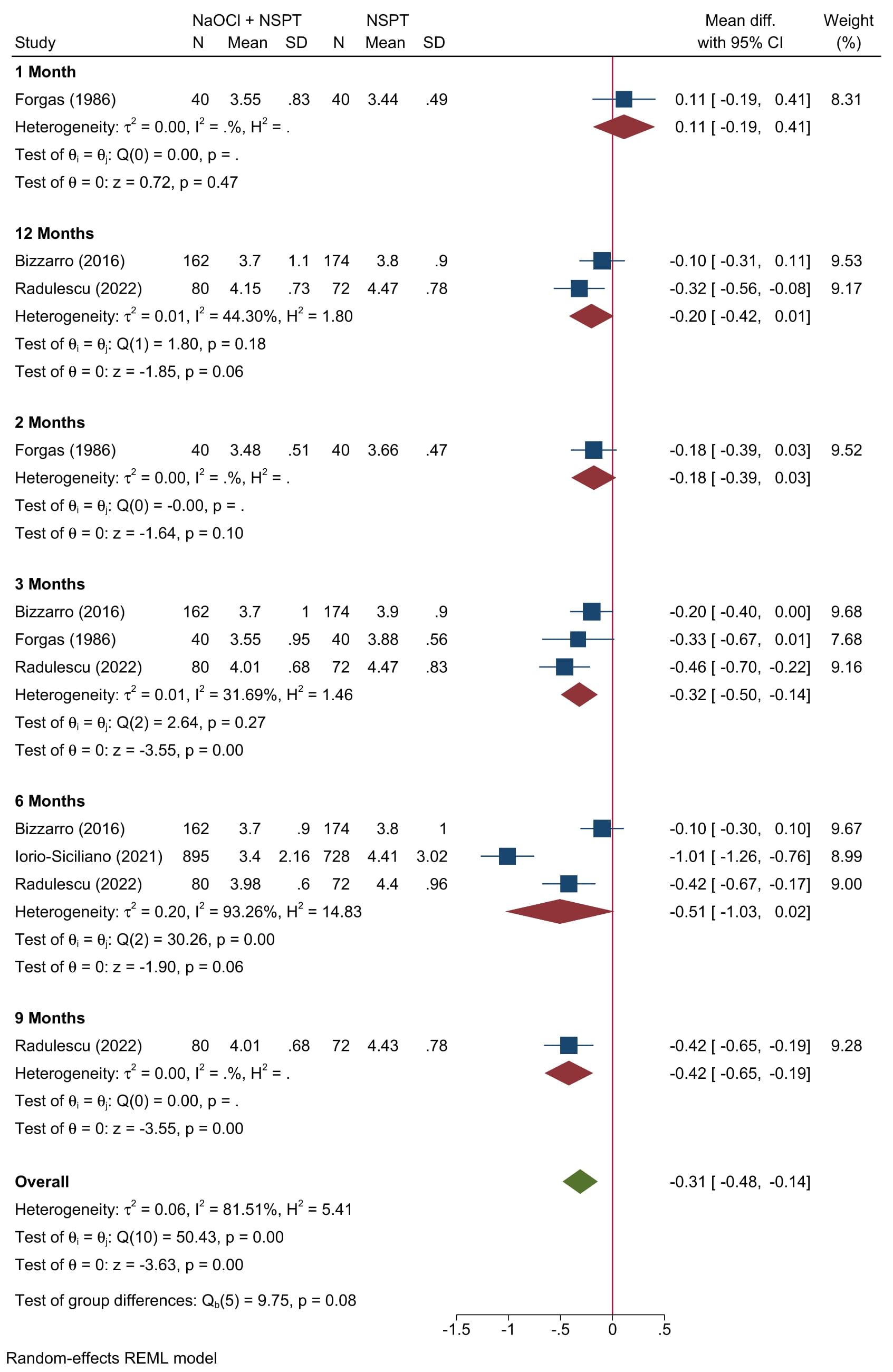

Supplement: Supplementary file 1 [file jcm-11-06593-s001.zip › Supp Figure S11.jpg]

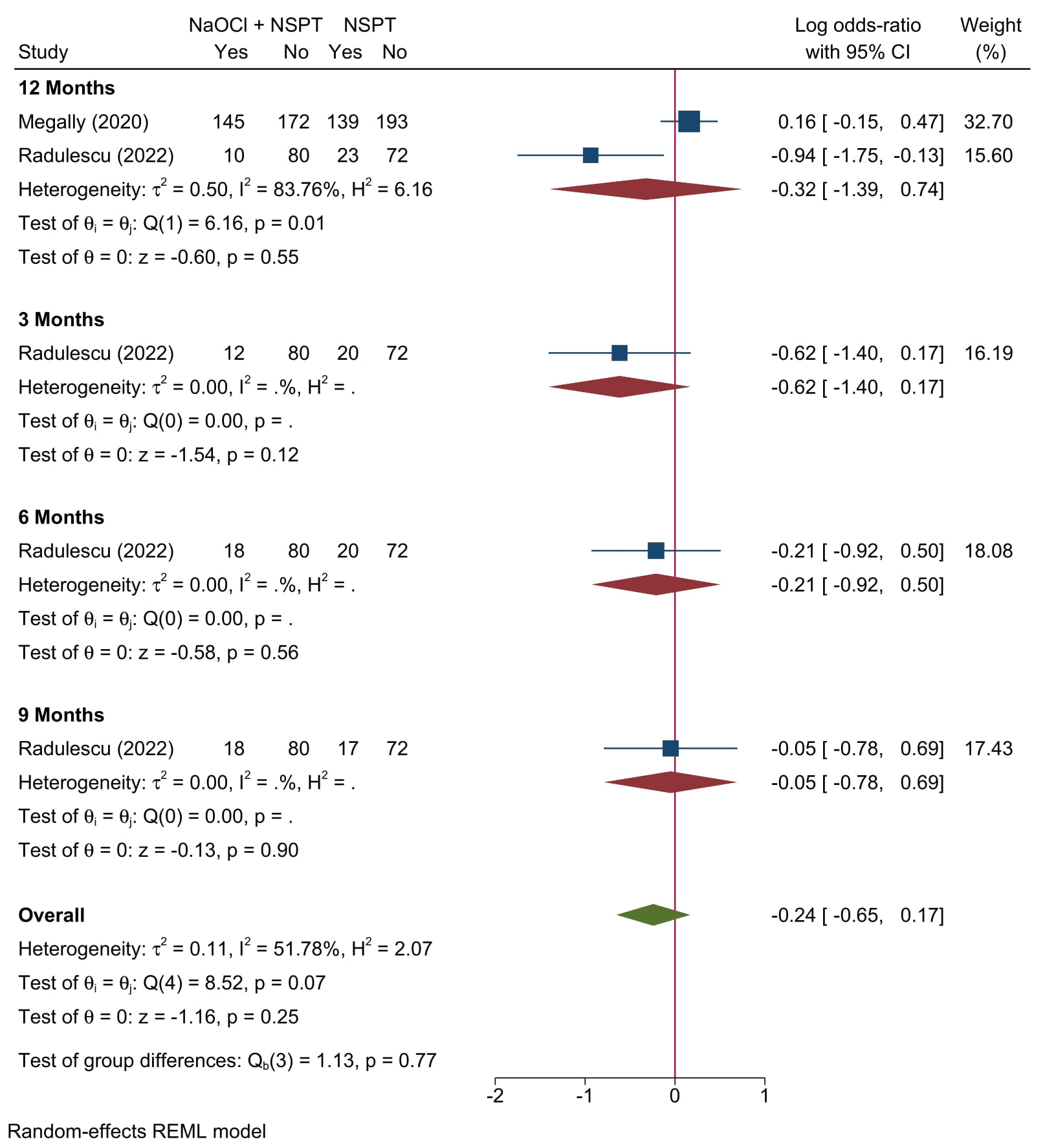

Supplement: Supplementary file 1 [file jcm-11-06593-s001.zip › Supp Figure S12.jpg]

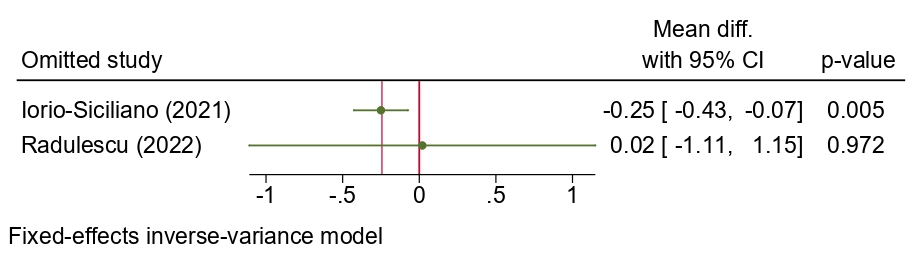

Supplement: Supplementary file 1 [file jcm-11-06593-s001.zip › Supp Figure S13.jpg]

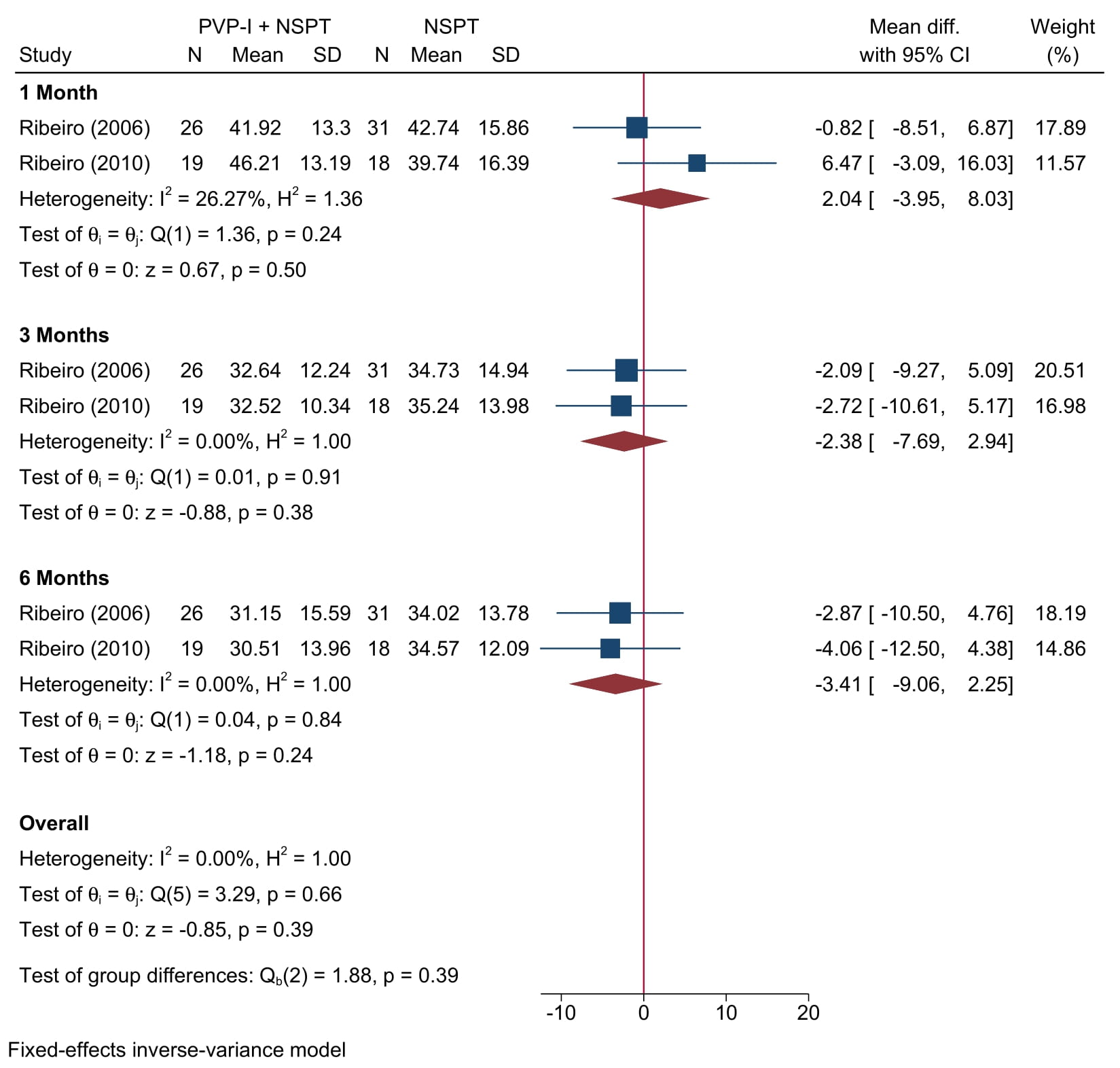

Supplement: Supplementary file 1 [file jcm-11-06593-s001.zip › Supp Figure S2.jpg]

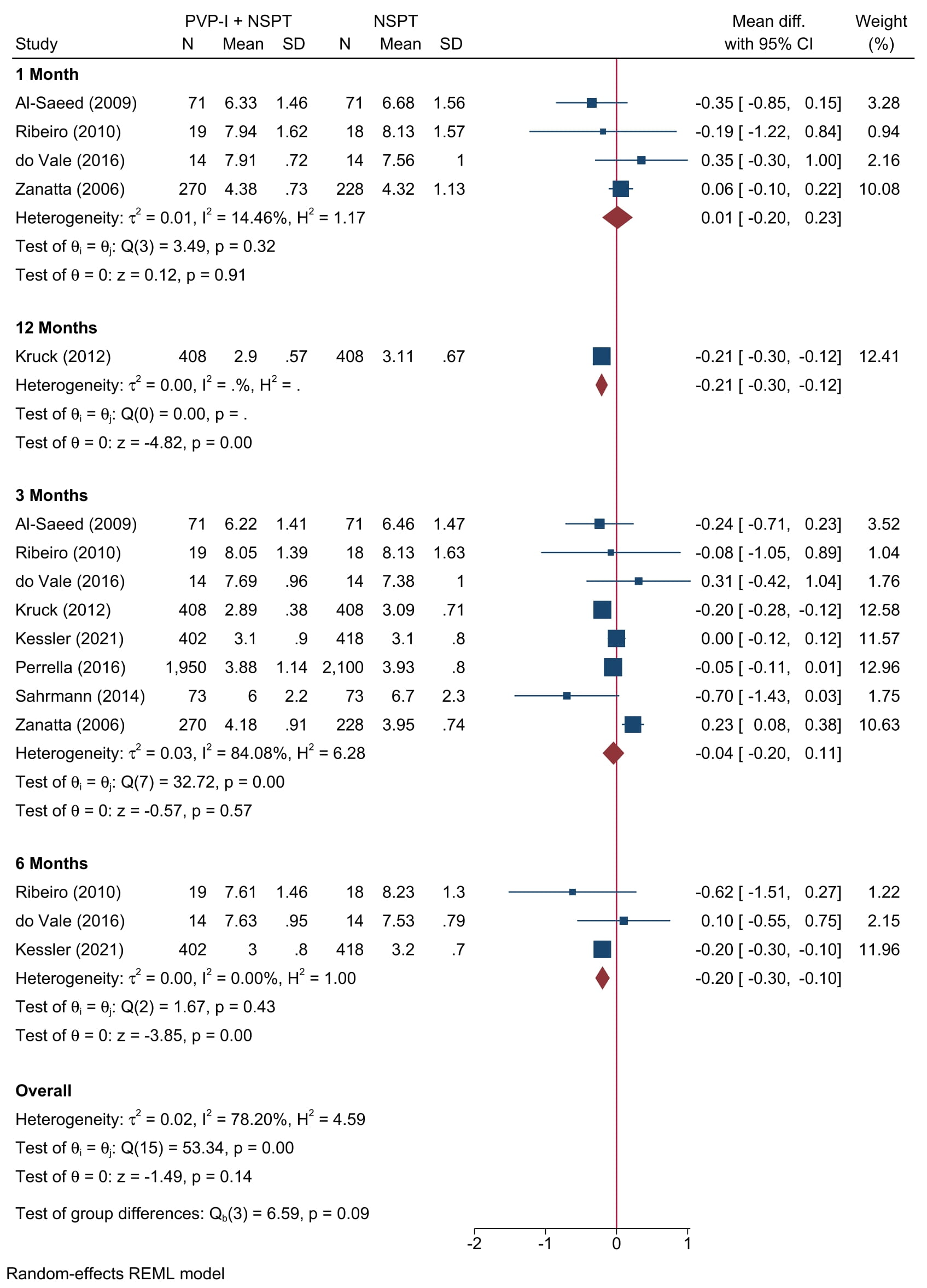

Supplement: Supplementary file 1 [file jcm-11-06593-s001.zip › Supp Figure S3.jpg]

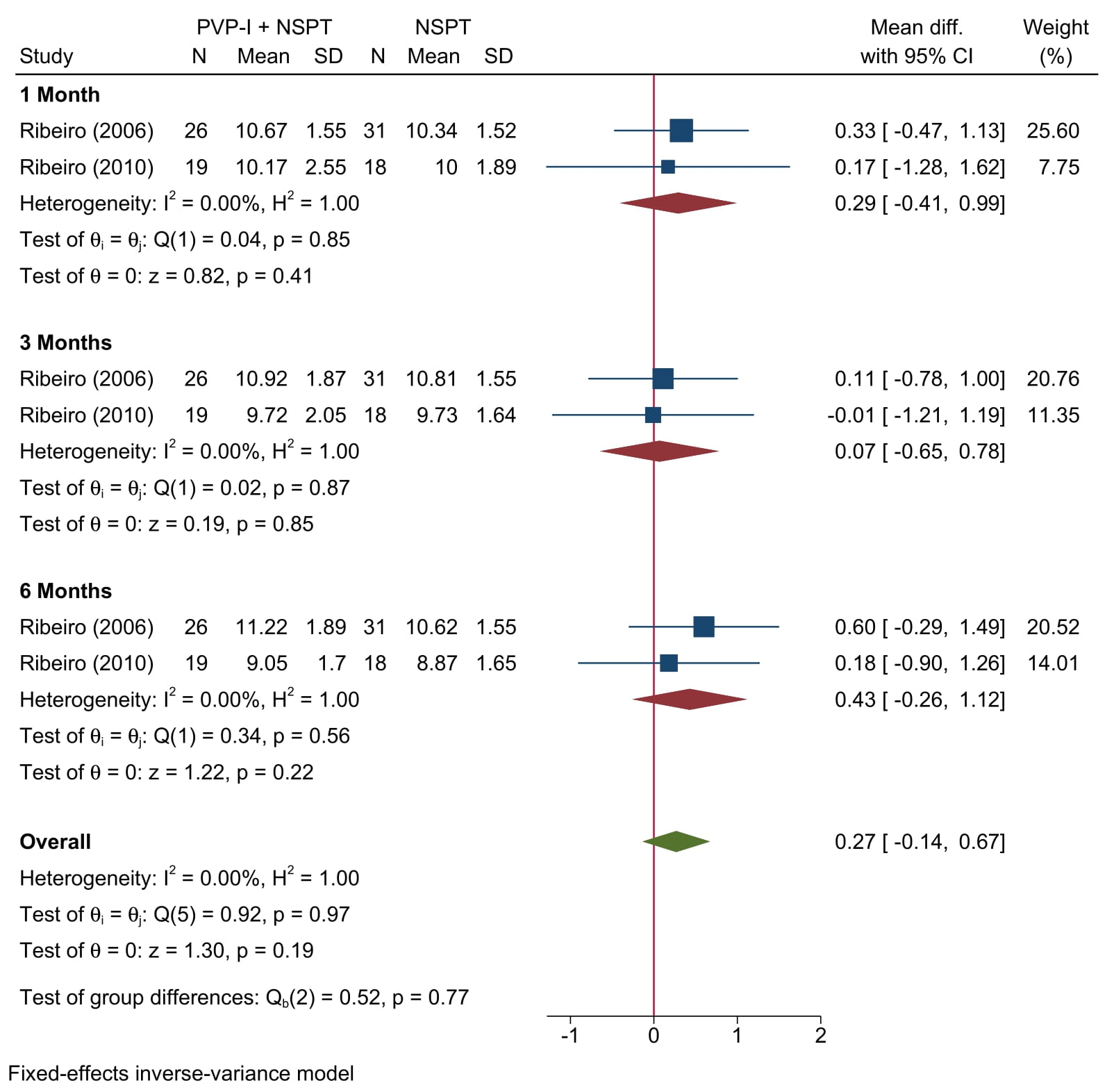

Supplement: Supplementary file 1 [file jcm-11-06593-s001.zip › Supp Figure S4.jpg]

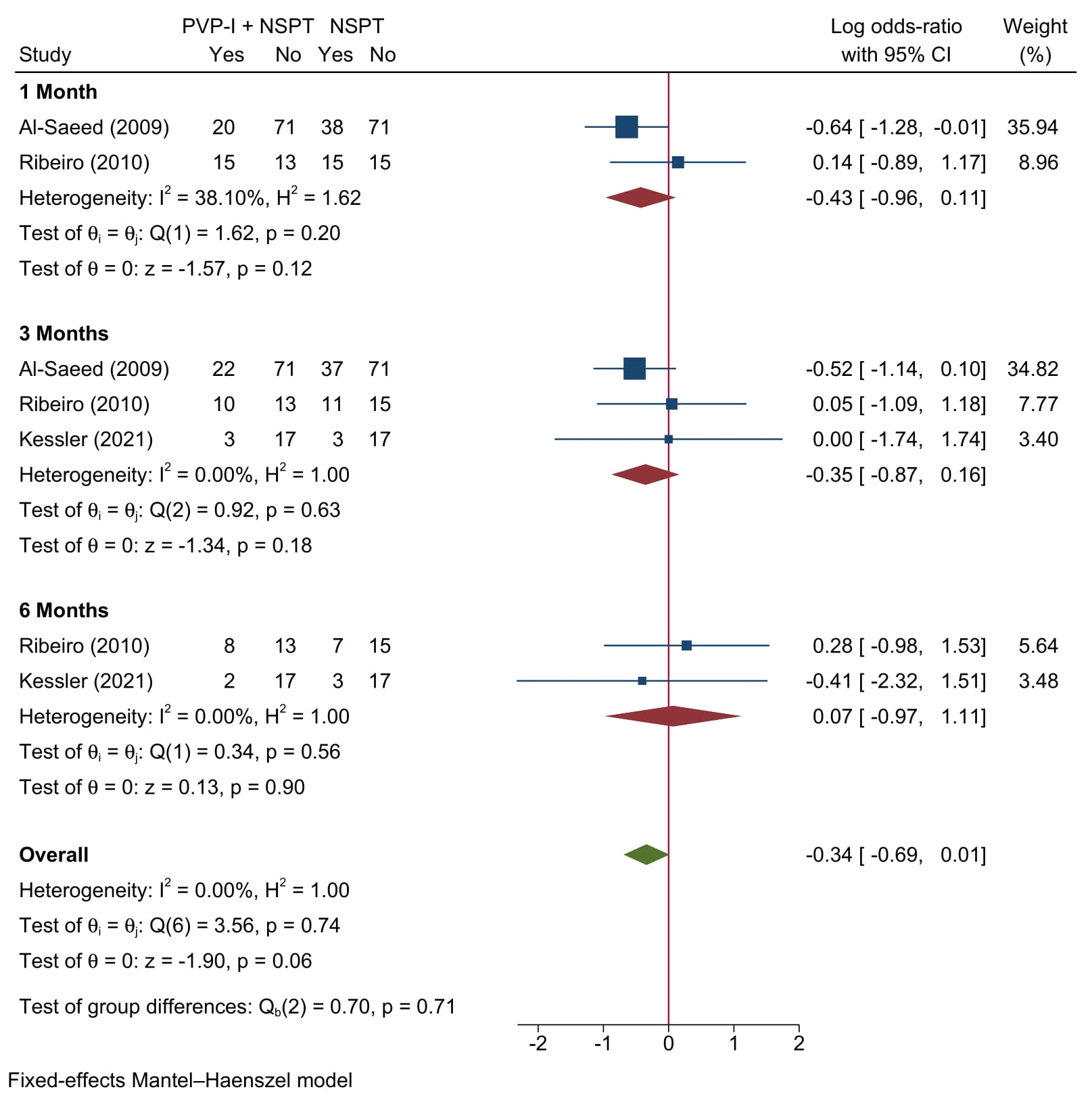

Supplement: Supplementary file 1 [file jcm-11-06593-s001.zip › Supp Figure S5.jpg]

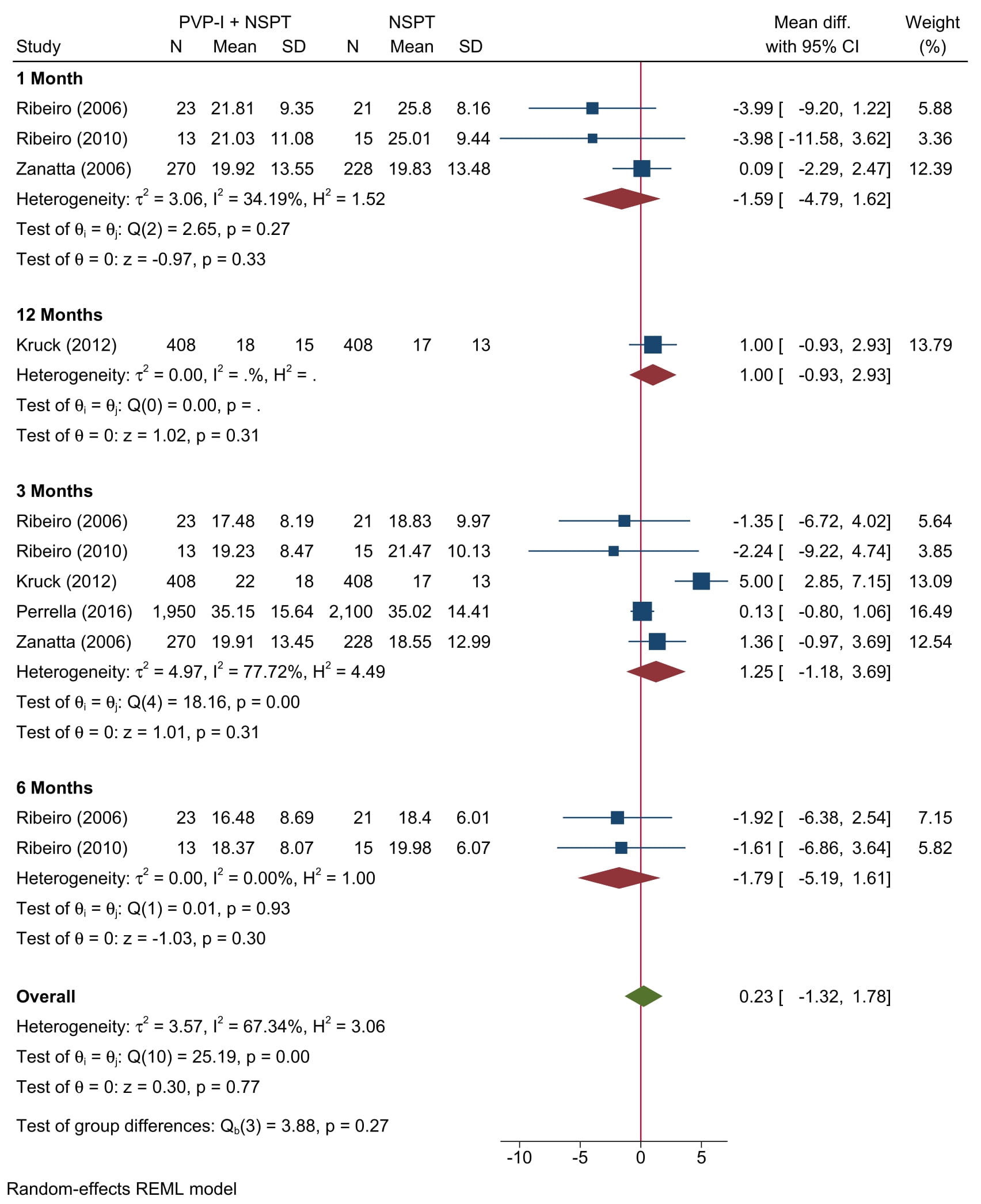

Supplement: Supplementary file 1 [file jcm-11-06593-s001.zip › Supp Figure S6.jpg]

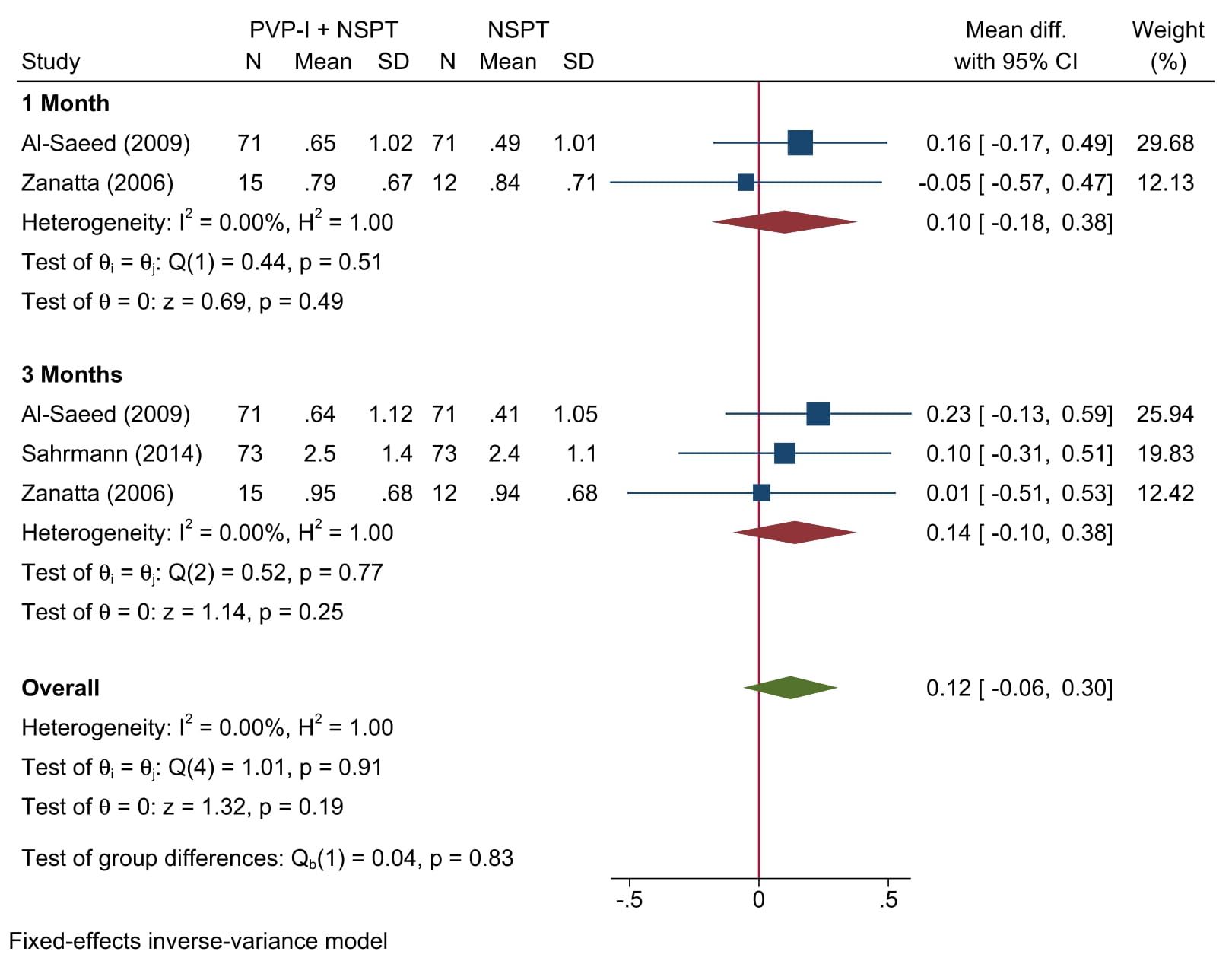

Supplement: Supplementary file 1 [file jcm-11-06593-s001.zip › Supp Figure S7.jpg]

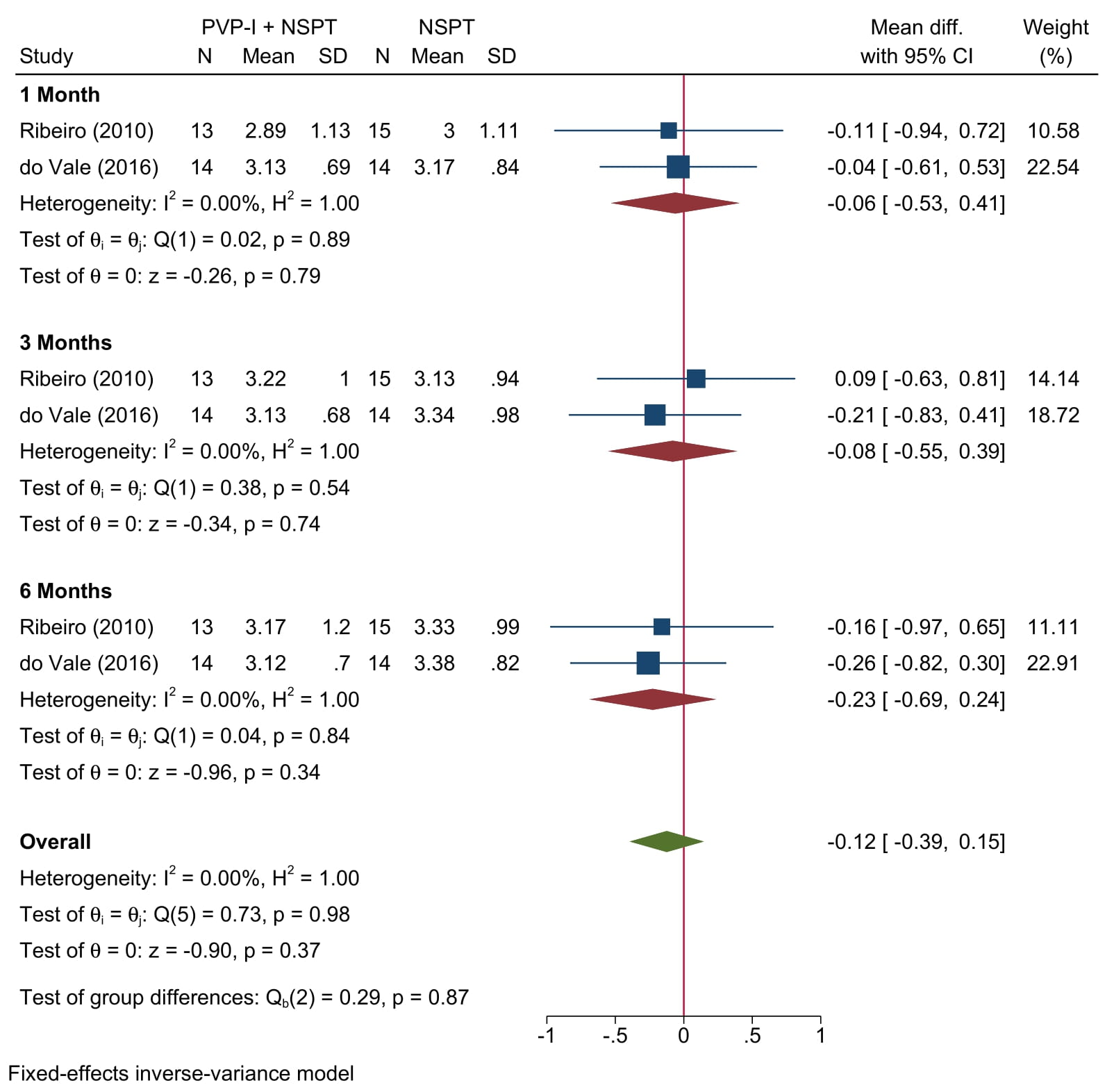

Supplement: Supplementary file 1 [file jcm-11-06593-s001.zip › Supp Figure S8.jpg]

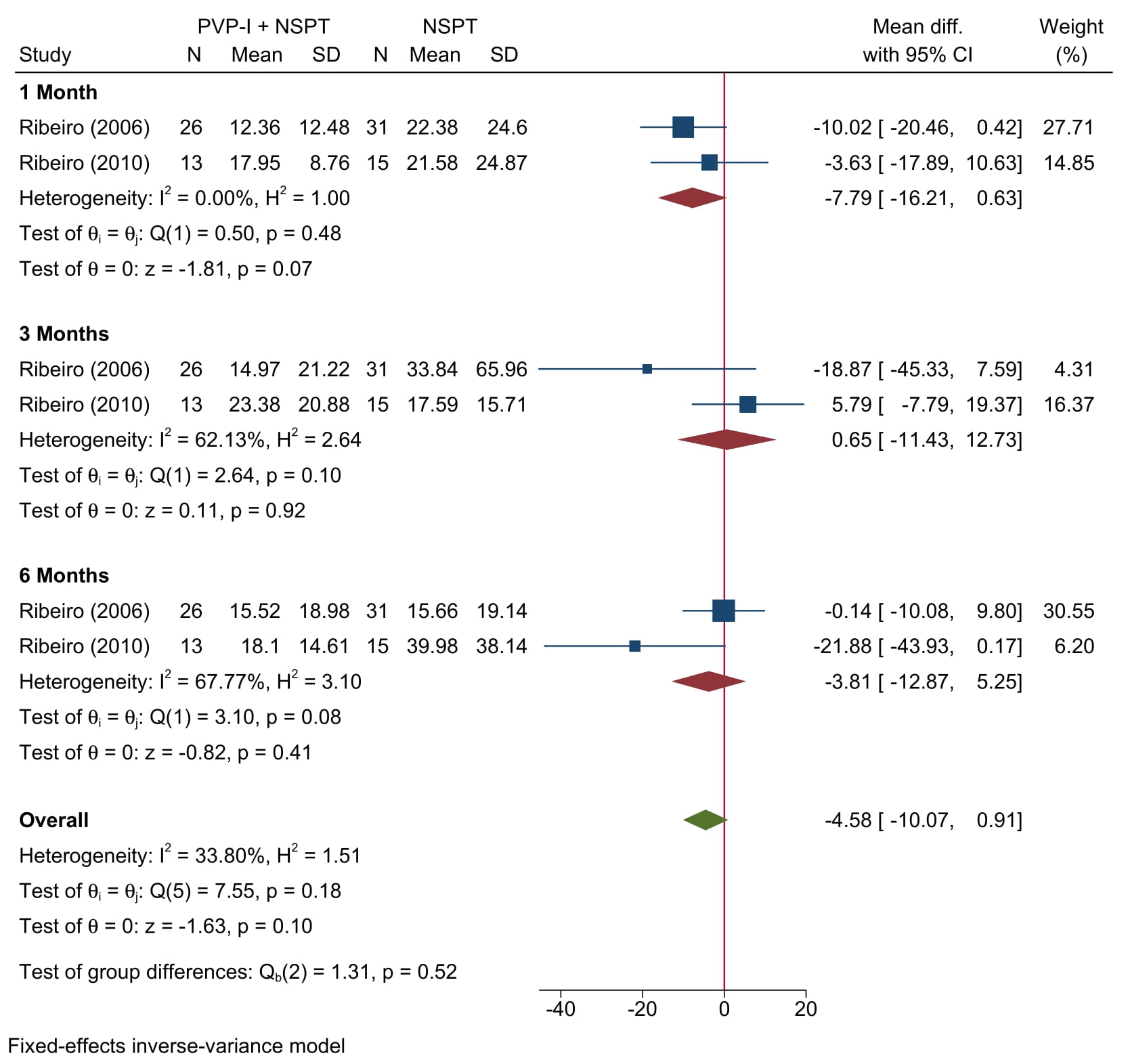

Supplement: Supplementary file 1 [file jcm-11-06593-s001.zip › Supp Figure S9.jpg]
